# Supplementary material for: Study on Flavonoids and Bioactivity Features of Pericarp of Citrus reticulata “Chachi” at Different Harvest Periods
Source: Plants (Basel). 2022 Dec 5;11(23):3390. doi: 10.3390/plants11233390 (PMC9737822; doi:10.3390/plants11233390)
Supplement: Supplementary file 1 [file plants-11-03390-s001.zip › plants-2032252-supplementary.pdf]

Table S1. The information of the detected metabolites

Compounds	Precursor ion	Ionization model	Formula	Class	A1	A2	A3	A4	A5	A6
Quercetin-O-rutinoside-hexose	771	[M-H]-	$C_{33}H_{40}O_{21}$	Flavonols	88900	233667	308667	300667	104067	461000
Isorhamnetin-3-O-rutinoside	623	[M-H]-	$C_{28}H_{32}O_{16}$	Flavonols	3553333	3023333	3233333	2210000	1142667	2585000
Kaempferol-3-O-glucoside-7-O-rhamnoside	595	[M+H]+	$C_{27}H_{30}O_{15}$	Flavonols	7993333	10900000	11023333	16166667	9763333	11600000
Quercetin-3-O-glucoside-7-O-rhamnoside	611	[M+H]+	$C_{27}H_{30}O_{16}$	Flavonols	590667	1007000	905667	567333	405333	451500
Quercetin 3-O-rhanosylgalactoside	611	[M+H]+	$C_{27}H_{30}O_{16}$	Flavonols	463667	842667	887000	1230000	790000	1138500
Gallic acid	169	[M-H]-	$C_7H_6O_5$	Flavanols	1350000	912333	781333	731667	1606667	444000
Hesperidin	609	[M-H]-	$C_{28}H_{34}O_{15}$	Dihydroflavone	1012000	662667	742333	984667	1110000	1235000
Formononetin	267	[M-H]-	$C_{16}H_{12}O_4$	Isoflavones	2473	785	1055	4347	8200	5190
Nobiletin	403	[M+H]+	$C_{21}H_{22}O_8$	Flavonoid	35033333	38700000	40266667	33266667	31666667	26900000
Quercitrin	449	[M+H]+	$C_{21}H_{20}O_{11}$	Flavonols	609333	589333	563000	605333	498000	589000
Naringin	579	[M-H]-	$C_{27}H_{32}O_{14}$	Dihydroflavone	106800	65600	111600	125533	104733	98050
Vitexin	431	[M-H]-	$C_{21}H_{20}O_{10}$	Flavonoid carbonoside	1913333	1140000	1966667	950667	639667	1160500
(+)-Gallocatechin	307	[M+H]+	$C_{15}H_{14}O_7$	Flavanols	103767	149767	239333	44503	7707	6345
Acacetin	285	[M+H]+	$C_{16}H_{12}O_5$	Flavonoid	4223	3323	4927	11257	15333	13975
Tangeretin	373	[M+H]+	$C_{20}H_{20}O_7$	Flavonols	11466667	8890000	10873333	10140000	9780000	7465000
Myricitrin	463	[M-H]-	$C_{21}H_{20}O_{12}$	Flavonols	408	985	540	1565	687	507
Eriodictyol 7-O-glucoside	451	[M+H]+	$C_{21}H_{22}O_{11}$	Dihydroflavone	27100	18433	26167	37133	21633	11455
Diosmetin	299	[M-H]-	$C_{16}H_{12}O_6$	Flavonoid	6450000	4070000	6260000	4946667	7623333	6080000
Rutin	609	[M-H]-	$C_{27}H_{30}O_{16}$	Flavonols	2496667	1678667	1518333	1267000	126633	2823500
Hyperin	463	[M-H]-	$C_{21}H_{20}O_{12}$	Flavonols	518333	413000	436333	413667	252333	343500
Eriodictyol	287	[M-H]-	$C_{15}H_{12}O_6$	Dihydroflavone	167333	82567	112000	158667	166333	268500
Isorhamnetin	315	[M-H]-	$C_{16}H_{12}O_7$	Flavonols	42133	32957	184000	107033	175867	474250

Apigenin 5-O-glucoside	433	[M+H]+	$C_{21}H_{20}O_{10}$	Flavonoid	187000	174000	279000	122600	82667	141700
Kaempferol 7-O-glucosdie	447	[M-H]-	$C_{21}H_{20}O_{11}$	Flavonols	157000	63100	112567	60267	32467	87650
Isoquercitrin	463	[M-H]-	$C_{21}H_{20}O_{12}$	Flavonols	90800	80433	72433	67800	46967	55250
Protocatechuic acid	153	[M-H]-	$C_7H_6O_4$	Flavanols	12300000	9790000	12800000	9823333	23566667	9065000
Hesperetin	301	[M-H]-	$C_{16}H_{14}O_6$	Dihydroflavone	360333	203733	174667	130233	181000	213000
4-Methylcatechol	123	[M-H]-	$C_7H_8O_2$	Flavanols	13867	36400	32700	22533	9087	6880
Poncirin(Isosakuranetin-7-neohesperidoside)	593	[M-H]-	$C_{28}H_{34}O_{14}$	Dihydroflavone	7903333	5376667	6096667	11663333	11036667	11080000
Pinobanksin	271	[M-H]-	$C_{15}H_{12}O_5$	Dihydroflavonol	1040333	360333	576000	1498333	774000	784500
3,7-Di-O-methylquercetin	329	[M-H]-	$C_{17}H_{14}O_7$	Flavonols	444667	152033	499333	269333	372333	1866500
Prunetin	283	[M-H]-	$C_{16}H_{12}O_5$	Isoflavones	389000	228333	388333	600333	863333	1104000
Kaempferin	431	[M-H]-	$C_{21}H_{20}O_{10}$	Flavonols	10150	53533	24367	38633	19000	6215
Tricetin	301	[M-H]-	$C_{15}H_{10}O_7$	Flavonoid	31333	15633	18667	24400	21267	15755
Rhamnetin(7-O-Methxyl Quercetin)	315	[M-H]-	$C_{16}H_{12}O_7$	Flavonols	436667	127800	150433	34900	101733	77550
Syringetin	345	[M-H]-	$C_{17}H_{14}O_8$	Flavonols	420000	430000	950667	343000	480000	808500
Isosakuranetin(4'-Methylnaringenin)	287	[M+H]+	$C_{16}H_{14}O_5$	Dihydroflavone	99633	60533	71600	185000	124333	108900
Kaempferol	285	[M-H]-	$C_{15}H_{10}O_6$	Flavonols	4990	3717	4003	7240	7927	13045
Apigenin 6,8-C-diglucoside	595	[M+H]+	$C_{27}H_{30}O_{15}$	Flavonoid	35266667	29366667	33566667	30600000	26200000	27900000
Dihydrokaempferol	287	[M-H]-	$C_{15}H_{12}O_6$	Dihydroflavonol	2910	2480	2567	1643	913	1372
Naringenin chalcone(4,2',4',6'-Tetrahydroxychalcone)	271	[M-H]-	$C_{15}H_{12}O_5$	Chalcones	1156667	384667	623667	1605667	882667	845000
Naringenin-7-O-glucoside	433	[M-H]-	$C_{21}H_{22}O_{10}$	Dihydroflavone	117367	67167	98800	134600	61033	48000
Tiliroside	593	[M-H]-	$C_{30}H_{26}O_{13}$	Flavonols	9123333	5936667	7110000	13750000	13533333	14005000
Isoschaftoside	565	[M+H]+	$C_{26}H_{28}O_{14}$	Flavonoid carbonoside	11900	8723	6743	7870	7713	6225
Orientin	449	[M+H]+	$C_{21}H_{20}O_{11}$	Flavonoid carbonoside	2656667	6163333	8050000	2906667	1222333	4370000
Sinensetin	373	[M+H]+	$C_{20}H_{20}O_7$	Flavonoid	14233333	14566667	15700000	13366667	12233333	10900000

Gossypitrin	463	[M-H]-	$C_{21}H_{20}O_{12}$	Flavonols	168333	100333	137367	108433	75533	53650
Isovitexin	431	[M-H]-	$C_{21}H_{20}O_{10}$	Flavonoid carbonoside	1780000	1155000	1966667	1031000	631000	1135000
Persicoside	477	[M-H]-	$C_{23}H_{26}O_{11}$	Dihydroflavone	212333	119633	132333	135000	150333	381000
5,7-Dihydroxy-3',4',5'-trimethoxyflavone	345	[M+H]+	$C_{18}H_{16}O_{7}$	Flavonoid	30066667	22666667	27433333	20533333	15966667	18950000
Eriocitrin	595	[M-H]-	$C_{27}H_{32}O_{15}$	Dihydroflavone	3133333	1723333	2650000	3946667	1366667	2500000
HoMoorientin	447	[M-H]-	$C_{21}H_{20}O_{11}$	Flavonoid carbonoside	1059333	2543333	4036667	1287333	706667	2622000
Schaftoside	565	[M+H]+	$C_{26}H_{28}O_{14}$	Flavonoid carbonoside	2086	2343	17840	11673	3150	1034
Diosmin	609	[M+H]+	$C_{28}H_{32}O_{15}$	Flavonoid	1706667	1743333	1733333	2386667	2313333	3075000
Tectochrysin	269	[M+H]+	$C_{16}H_{12}O_{4}$	Flavonoid	6213	4593	6230	4777	1443	2025
Phloretin 2'-O-glucoside	435	[M-H]-	$C_{21}H_{24}O_{10}$	Isoflavones	43800	38033	53033	37833	49533	152800
Astragalin	447	[M-H]-	$C_{21}H_{20}O_{11}$	Flavonols	189667	51333	105733	56300	27433	89950
8-Methoxychrysin(Wogonin)	285	[M+H]+	$C_{16}H_{12}O_{5}$	Flavonoid	8187	8020	9110	13953	19233	16100
Luteolin O-feruloylhexoside	625	[M+H]+	$C_{28}H_{32}O_{16}$	Flavonoid	18900	21367	16300	20633	16500	11850
Luteolin O-hexosyl-O-pentoside	581	[M+H]+	$C_{26}H_{28}O_{15}$	Flavonoid	19567	23067	12570	12967	5170	6120
Luteolin O-sinapoylhexoside	655	[M+H]+	$C_{32}H_{30}O_{15}$	Flavonoid	473	1797	6520	5073	7710	10300
Chrysoeriol O-sinapoylhexoside	669	[M+H]+	$C_{33}H_{32}O_{15}$	Flavonoid	13400	56367	175333	130600	183067	396700
Luteolin 3',7-di-O-glucoside	611	[M+H]+	$C_{27}H_{30}O_{16}$	Flavonoid	16433	49400	52433	22933	19907	58650
Chrysoeriol O-hexosyl-O-rutinoside	771	[M+H]+	$C_{34}H_{42}O_{20}$	Flavonoid	926000	1032333	717333	931333	733333	1165000
Chrysoeriol O-hexosyl-O-hexoside	625	[M+H]+	$C_{28}H_{32}O_{16}$	Flavonoid	34333	50833	62200	37400	29267	42850
Chrysoeriol O-malonylhexoside	549	[M+H]+	$C_{25}H_{24}O_{14}$	Flavonoid	1276667	2023333	2533333	1663333	518333	583500
Apigenin 6-C-hexosyl-8-C-hexosyl-O-hexoside	757	[M+H]+	$C_{33}H_{40}O_{20}$	Flavonoid	20000	25700	40233	53000	69333	51950
8-C-Hexosyl-hesperetin O-hexoside	627	[M+H]+	$C_{28}H_{34}O_{16}$	Flavonoid carbonoside	23637	46400	58500	70833	57733	123500
Chrysoeriol 6-C-hexoside	787	[M+H]+	$C_{34}H_{42}O_{21}$	Flavonoid	15633	20400	26067	25367	29900	26850

8-C-hexoside-O-hexoside										
C-Hexosyl-luteolin O-hexoside	611	[M+H]+	$C_{27}H_{30}O_{16}$	Flavonoid carbonoside	146000	247667	283667	217333	331333	400000
6-C-Hexosyl chrysoeriol O-hexoside	625	[M+H]+	$C_{28}H_{32}O_{16}$	Flavonoid carbonoside	110600	73900	97267	79133	139000	134500
6-C-Hexosyl-luteolin O-hexoside	611	[M+H]+	$C_{27}H_{30}O_{16}$	Flavonoid carbonoside	28867	60800	58367	68667	222667	344500
6-C-Hexosyl-apigenin O-hexosyl-O-hexoside	757	[M+H]+	$C_{33}H_{40}O_{20}$	Flavonoid carbonoside	12450	16633	25433	31067	48333	29550
Chrysoeriol 6-C-hexoside	463	[M+H]+	$C_{22}H_{22}O_{11}$	Flavonoid	12967	23433	28667	185000	878333	1169500
6-C-Hexosyl luteolin O-pentoside	581	[M+H]+	$C_{26}H_{28}O_{15}$	Flavonoid carbonoside	36833	138967	164800	61900	74133	153800
6-C-Hexosyl-hesperetin O-hexoside	627	[M+H]+	$C_{28}H_{34}O_{16}$	Flavonoid carbonoside	97167	112000	111133	56400	57033	43250
Isorhamnetin C-hexoside	479	[M+H]+	$C_{22}H_{22}O_{12}$	Flavonols	24933	23600	21067	8633	5983	6875
C-Hexosyl-apigenin O-pentoside	565	[M+H]+	$C_{26}H_{28}O_{14}$	Flavonoid carbonoside	3786667	5030000	8693333	3133333	3966667	5655000
Di-C,C-hexosyl-apigenin	595	[M+H]+	$C_{27}H_{30}O_{15}$	Flavonoid	94300	119167	202333	74667	92667	121200
Luteolin 8-C-hexosyl-O-hexoside	611	[M+H]+	$C_{27}H_{30}O_{16}$	Flavonoid	227000	312667	267333	126900	21540	194850
6-C-Hexosyl-apigenin O-sinapoylhexoside	801	[M+H]+	$C_{38}H_{40}O_{19}$	Flavonoid carbonoside	27000	12567	10790	10260	7797	12000
8-C-Hexosyl-apigenin O-feruloylhexoside	771	[M+H]+	$C_{37}H_{38}O_{18}$	Flavonoid carbonoside	24367	14200	20900	23567	27867	27800
8-C-Hexosyl-apigenin O-sinapoylhexoside	801	[M+H]+	$C_{38}H_{40}O_{19}$	Flavonoid carbonoside	3937	5847	5267	6613	9540	7315
Chrysoeriol C-hexoside	463	[M+H]+	$C_{22}H_{22}O_{11}$	Flavonoid	56800	37800	36500	22567	15900	20300
Luteolin C-hexosyl-O-rhamnoside O-hexoside	757	[M+H]+	$C_{33}H_{40}O_{20}$	Flavonoid	6603	35767	26267	17800	11147	20150
Tricin 7-O-hexosyl-O-hexoside	655	[M+H]+	$C_{29}H_{34}O_{17}$	Flavonoid	369667	370000	503000	525000	653667	539500

Tricin 4'-O-(syringyl alcohol)ether 5-O-hexoside	659	[M+H]+	$C_{32}H_{34}O_{15}$	Flavonoid	23300	18233	14467	15700	16567	13600
Tricin O-malonylhexoside	579	[M+H]+	$C_{26}H_{26}O_{15}$	Flavonoid	2430000	3163333	2896667	1733333	607000	446500
Tricin 7-O-feruloylhexoside	669	[M+H]+	$C_{33}H_{32}O_{15}$	Flavonoid	55933	41767	87200	79267	70433	72400
Tricin 7-O-hexoside	493	[M+H]+	$C_{23}H_{24}O_{12}$	Flavonoid	9206667	6953333	7180000	5523333	4220000	4955000
Tricin O-sinapoylhexoside	699	[M+H]+	$C_{34}H_{34}O_{16}$	Flavonoid	7017	13500	42433	54733	61533	44000
Tricin O-hexosyl-O-syringin alcohol	659	[M+H]+	$C_{32}H_{34}O_{15}$	Flavonoid	52033	41167	42200	50500	41633	46200
Hesperetin O-hexosyl-O-hexoside	625	[M-H]-	$C_{28}H_{34}O_{16}$	Dihydroflavonol	6087	6343	7453	7933	5520	16000
Hesperetin O-Glucuronic acid	477	[M-H]-	$C_{23}H_{26}O_{11}$	Dihydroflavonol	8806	43433	40333	47967	50533	50900
Chrysoeriol C-pentosyl-O-hexosyl-O-hexoside	755	[M-H]-	$C_{33}H_{40}O_{20}$	Flavonoid	34933	75567	125433	48900	59667	213450
Hesperetin O-malonylhexoside	549	[M-H]-	$C_{25}H_{26}O_{14}$	Dihydroflavonol	133000	124667	109667	149667	408000	321000
Apigenin O-hexosyl-O-rutinoside	739	[M-H]-	$C_{33}H_{40}O_{19}$	Flavonoid	28100	10963	19200	23667	17133	19050
Chrysoeriol O-hexosyl-O-hexosyl-O-Glucuronic acid	799	[M-H]-	$C_{34}H_{40}O_{22}$	Flavonoid	335667	245667	177000	92400	54667	95450
Chrysoeriol 5-O-hexoside	461	[M-H]-	$C_{22}H_{22}O_{11}$	Flavonoid	165000	149000	174667	122333	99200	120050
Chrysoeriol O-acetylhexoside	503	[M-H]-	$C_{24}H_{24}O_{12}$	Flavonoid	3533333	3916667	4440000	3296667	1253333	998500
Chrysoeriol 7-O-rutinoside	607	[M-H]-	$C_{28}H_{32}O_{15}$	Flavonoid	5523333	4086667	4086667	6686667	6506667	9955000
Apigenin 7-O-glucoside(Cosmosiin)	431	[M-H]-	$C_{21}H_{20}O_{10}$	Flavonoid	6047	5243	8397	19310	36533	48150
Chrysoeriol 7-O-hexoside	461	[M-H]-	$C_{22}H_{22}O_{11}$	Flavonoid	2653333	1624667	2533333	940667	289333	1066000
Isorhamnetin O-acetyl-hexoside	519	[M-H]-	$C_{24}H_{24}O_{13}$	Flavonols	14900	18400	20867	8280	3203	5245
Luteolin C-hexoside	447	[M-H]-	$C_{21}H_{20}O_{11}$	Flavonoid	676667	2059333	2953333	1024667	505333	1769500
Quercetin O-acetylhexoside	505	[M-H]-	$C_{23}H_{22}O_{13}$	Flavonoid	22733	89033	134733	31067	17200	11950
Tricin O-malonyl rhamnoside	561	[M-H]-	$C_{26}H_{26}O_{14}$	Flavonoid	1233	1070	2610	2300	8717	14540
Tricin O-saccharic acid	521	[M-H]-	$C_{23}H_{22}O_{14}$	Flavonoid	334000	454667	575667	816000	556333	1170000
Tricin 5-O-hexoside	491	[M-H]-	$C_{23}H_{24}O_{12}$	Flavonoid	48767	23167	27833	24167	42000	37800
Tricin 5-O-rutinoside	637	[M-H]-	$C_{29}H_{34}O_{16}$	Flavonoid	255333	117233	135667	116800	36133	95250
Tricin O-eudesmic acid	523	[M-H]-	$C_{27}H_{24}O_{11}$	Flavonoid	6247	98767	135233	161567	76500	14500

Di-O-methylquercetin	329	[M-H]-	$C_{17}H_{14}O_7$	Flavonols	13733333	12426667	12200000	11933333	13600000	11315000
Hesperetin 7-O-neohesperidoside(Neohesperidin)	609	[M-H]-	$C_{28}H_{34}O_{15}$	Dihydroflavone	1106333	764333	780667	955667	1216667	1320000
Luteolin	285	[M-H]-	$C_{15}H_{10}O_6$	Flavonoid	15967	29600	50333	68700	111800	260000
Methyl gallate	183	[M-H]-	$C_8H_8O_5$	Flavanols	23900	41700	42000	43800	39700	30850
Kaempferol 7-O-rhamnoside	431	[M-H]-	$C_{21}H_{20}O_{10}$	Flavonols	11020	59800	24067	37000	20267	7420
Apigenin 7-rutinoside(Isorhoifolin)	579	[M+H]+	$C_{27}H_{30}O_{14}$	Flavonoid	16033333	9506667	12123333	23733333	12633333	13250000
Kaempferol 3-O-rutinoside(Nicotiflorin)	593	[M-H]-	$C_{27}H_{30}O_{15}$	Flavonols	4330000	3140000	3883333	6223333	3390000	7810000
Naringenin	271	[M-H]-	$C_{15}H_{12}O_5$	Dihydroflavone	1094333	362667	590333	1580000	822667	861000
Isorhamnetin 3-O-neohesperidoside	625	[M+H]+	$C_{28}H_{32}O_{16}$	Flavonols	120667	244667	277667	201333	186667	201200
Hesperetin 5-O-glucoside	463	[M-H]-	$C_{22}H_{24}O_{11}$	Dihydroflavonol	70500	80133	93867	45200	38033	66150
Kaempferol 3-O-robinobioside(Biorobin)	593	[M-H]-	$C_{27}H_{30}O_{15}$	Flavonols	4593333	3223333	3603333	6186667	3580000	7285000
Isohemiphloin	433	[M-H]-	$C_{21}H_{22}O_{10}$	Flavonoid carbonoside	86100	55567	83667	48267	29367	43850
Isovitexin 7-O-glucoside(Saponarin)	593	[M-H]-	$C_{27}H_{30}O_{15}$	Flavonoid carbonoside	408333	328333	346667	305000	284667	279500
Luteolin 7-O-glucoside(Cynaroside)	449	[M+H]+	$C_{21}H_{20}O_{11}$	Flavonoid	819000	518000	343667	366667	1653333	1351000
Protocatechuic aldehyde	139	[M+H]+	$C_7H_6O_3$	Flavanols	90200	77833	95633	72167	96767	83550
Naringenin chalcone	273	[M+H]+	$C_{15}H_{12}O_5$	Chalcones	402333	165667	243333	705333	378667	392000
Vitexin 2''-O-β-L-rhamnoside	579	[M+H]+	$C_{27}H_{30}O_{14}$	Flavonoid carbonoside	219333	220667	385667	156333	158733	252000
Biochanin A	285	[M+H]+	$C_{16}H_{12}O_5$	Isoflavones	8237	7927	11497	13593	18033	13510
2'-Hydroxygenistein	287	[M+H]+	$C_{15}H_{10}O_6$	Isoflavones	372000	222667	219000	447667	387667	355500
Butin	273	[M+H]+	$C_{15}H_{12}O_5$	Dihydroflavone	446333	168000	241333	647000	383333	409500
Formononetin 7-O-glucoside(Ononin)	431	[M+H]+	$C_{22}H_{22}O_9$	Isoflavones	14000	9630	15157	8990	9647	8945
Syringaldehyde	315	[M-H]-	$C_{16}H_{12}O_7$	Flavonoid	25033	63167	79933	80700	143867	395000
Natsudaidain	417	[M-H]-	$C_{21}H_{22}O_9$	Flavonols	23133333	18066667	16300000	10383333	12033333	8075000
Sexangularetin	315	[M-H]-	$C_{16}H_{12}O_7$	Flavonols	22400	13500	29733	20033	11193	9275

Bioquercetin	609	[M-H]-	$C_{27}H_{30}O_{16}$	Flavonols	3323333	2246667	2168333	1747000	164000	2943500
Cynaroside	447	[M-H]-	$C_{21}H_{20}O_{11}$	Flavonoid	1987	5900	8033	14743	1341	1526
Luteolin-7-O-β-D-glucuronide	447	[M-H]-	$C_{21}H_{20}O_{11}$	Flavonoid	31433	57567	95067	35233	29000	89600
Tetahydroxy-flavone-7-O-β-D-glucuronide	461	[M-H]-	$C_{21}H_{18}O_{12}$	Flavonoid	68867	56900	65200	49200	33167	33250
Luteolin-7-O-β-D-rutinoside	593	[M-H]-	$C_{27}H_{30}O_{15}$	Flavonoid	8333333	7096667	7400000	6976667	6496667	5955000
Hispidulin	301	[M+H]+	$C_{16}H_{12}O_{6}$	Flavonoid	4736667	3086667	5496667	7416667	10060000	19500000
Ladanein	315	[M+H]+	$C_{17}H_{14}O_{6}$	Flavonoid	159333	407567	1503000	2423333	1614667	6682000
Jaceosidin	331	[M+H]+	$C_{17}H_{14}O_{7}$	Anthocyanins	7460000	3806667	5160000	2490000	2476667	3400000
Eupatilin	345	[M+H]+	$C_{18}H_{16}O_{7}$	Flavonoid	19600000	16800000	19266667	14933333	13566667	13100000
5-Hydroxy-6,7,3',4'-tetramethoxyflavone	359	[M+H]+	$C_{19}H_{18}O_{7}$	Flavonoid	1143333	1430000	1536667	1390000	1089333	1380000
5,7,4',5'-Tetrahydro-3',6-dimethoxyflavone	347	[M+H]+	$C_{17}H_{14}O_{8}$	Flavonoid	699667	591333	717000	500667	455333	440000
Eupatilin 3-glucoside	507	[M+H]+	$C_{24}H_{26}O_{12}$	Flavonoid	11586667	9356667	7703333	7426667	5096667	4530000
5,7,4′-Trimethoxyflavone	313	[M+H]+	$C_{18}H_{16}O_{5}$	Flavonoid	266333	223667	205333	180333	372333	243000
Monohydroxy-trimethoxyflavone	329	[M+H]+	$C_{18}H_{16}O_{6}$	Other Flavonoids	3183333	1936667	2446667	1558667	897667	812000
Tetramethyl-O-isoscutellarein	343	[M+H]+	$C_{19}H_{18}O_{6}$	Flavonoid	804000	783667	804667	766000	724667	596000
7-Hydroxy-3,5,6,8-tetramethoxyflavone	359	[M+H]+	$C_{19}H_{18}O_{7}$	Flavonoid	13333333	13700000	17000000	11700000	10646667	8645000
5-Hydroxy-6,7,8,3',4'-pentamethoxyflavone	389	[M+H]+	$C_{20}H_{20}O_{8}$	Flavonoid	11733333	8056667	8573333	6966667	7603333	5335000
5,6,7,8,3′,4′-Hexamethoxyflavanone	405	[M+H]+	$C_{21}H_{24}O_{8}$	Dihydroflavone	1610000	1426333	1540000	1025333	1343333	1360000
Monohydroxy-hexamethoxyflavone	419	[M+H]+	$C_{21}H_{22}O_{9}$	Other Flavonoids	26866667	26033333	27933333	25200000	20333333	23900000
Apigenin-8-C-glucoside	433	[M+H]+	$C_{21}H_{20}O_{10}$	Flavonoid carbonoside	2486667	2006667	3413333	1616667	839667	1801000
3,5,6,7,8,3′,4′-Heptamethoxyflavone	433	[M+H]+	$C_{22}H_{24}O_{9}$	Flavonols	1326667	1333333	1316667	1143333	1059333	959000
Diosmetin-6-C-glucoside	463	[M+H]+	$C_{22}H_{22}O_{11}$	Flavonoid	20933333	20333333	21000000	16033333	11333333	14350000
Luteolin-6,8-di-C-glucoside	611	[M+H]+	$C_{27}H_{30}O_{16}$	Flavonoid	7866667	12213333	12856667	9910000	7533333	9910000
Chysoeriol-6,8-di-C-glucoside	625	[M+H]+	$C_{28}H_{32}O_{16}$	Flavonoid	42166667	40566667	46833333	33766667	27933333	32000000

				carbonoside						
Limocitrin-3-O-(3-hydroxy-3-methylglutarate)-glucoside	653	[M+H]+	$C_{29}H_{32}O_{17}$	Flavonols	2186667	970333	888000	378667	289667	412500
Natsudaidain??3-O-(3-hydroxy-3-methylglutarate)-glucoside)	725	[M+H]+	$C_{33}H_{40}O_{18}$	Flavonols	1080000	713333	568333	361667	341000	187500
Natsudaidain??3-O-(5-glucosyl-3-hydroxy-3-methylglutarate)-glucoside)	887	[M+H]+	$C_{39}H_{50}O_{23}$	Flavonols	1556667	1923333	3460000	3536667	4486667	2010000
5,2'-Dihydroxy-7,8-dimethoxyflavone	315	[M+H]+	$C_{17}H_{14}O_{6}$	Flavonoid	70367	74700	86433	98433	89867	63600
5,2'-Dihydroxy-7,8-dimethoxyflavone glycosides	477	[M+H]+	$C_{23}H_{24}O_{11}$	Flavonoid	158667	150333	193333	251000	278000	470000
Malonyglygenistin	519	[M+H]+	$C_{24}H_{22}O_{13}$	Isoflavones	7197	12100	18973	14547	5243	5800
Apigenin-6-C-glucose-8-xylcose	565	[M+H]+	$C_{26}H_{28}O_{14}$	Flavonoid carbonoside	730333	896333	1636667	621000	688333	914000
Apigenin-6-C-2-glucuronylxyloside	579	[M+H]+	$C_{26}H_{26}O_{15}$	Flavonoid carbonoside	83967	110967	174667	68067	56300	107700
Luteolin 7-O-β-D-glucosyl-6-C-α-L-arabinose	581	[M+H]+	$C_{26}H_{28}O_{15}$	Flavonoid	89367	335333	467333	134233	168167	370500
Luteolin-6-C-5-glucuronylxyloside	595	[M+H]+	$C_{26}H_{26}O_{16}$	Flavonoid	93467	58667	78700	47467	41200	43850
Luteolin-6-C-2-glucuronylglucoside	625	[M+H]+	$C_{27}H_{28}O_{17}$	Flavonoid	13433333	13800000	14200000	10376667	7946667	11355000
3′,4′,7-Trihydroxyflavone	271	[M+H]+	$C_{15}H_{10}O_{5}$	Flavonoid	231333	68667	19877	5760	16733	12960
Naringenin 7-O-(2-β-Dapiofuranosyl)-β-D-glucopyranoside	551	[M+H]+	$C_{26}H_{30}O_{13}$	Dihydroflavone	1143	1815	1890	2089	179333	231500
Ononin	431	[M+H]+	$C_{22}H_{22}O_{9}$	Isoflavones	77633	64000	87100	46300	49100	41800
Genistein 8-C-apiosyl(1→6)glucoside	565	[M+H]+	$C_{26}H_{28}O_{14}$	Flavonoid carbonoside	3210000	3926667	6956667	2373333	3097667	4435000
Genistein 8-C-glucoside	433	[M+H]+	$C_{21}H_{20}O_{10}$	Flavonoid carbonoside	4766667	3166667	5316667	2600000	1385333	3160000
Pratensein	301	[M+H]+	$C_{16}H_{12}O_{6}$	Isoflavones	5353333	2930000	5850000	7086667	10623333	19550000
Apigenin	271	[M+H]+	$C_{15}H_{10}O_{5}$	Flavonoid	26767	12967	24300	39600	33367	47650
Diosmetin-7-O-galactoside	463	[M+H]+	$C_{22}H_{22}O_{11}$	Flavonoid	20900000	11176667	16270000	7886667	4053333	13030000

Isoquercitrin(Quercetin 3-O-β-D-glucoside)	465	[M+H]+	$C_{21}H_{20}O_{12}$	Flavonols	586333	793000	669000	432000	106133	481000
Diosmetin-7-O-(6'-O-malonyl)-β-D-glucoside	549	[M+H]+	$C_{25}H_{24}O_{14}$	Flavonoid	43433	53967	75467	54600	14500	18600
Acacetin-7-O-rutinoside	593	[M+H]+	$C_{28}H_{32}O_{14}$	Flavonoid	167667	69367	71267	206000	182333	287500
Luteolin-7-O-rutinoside	595	[M+H]+	$C_{27}H_{30}O_{15}$	Flavonoid	7826667	10173333	11566667	13933333	10453333	9480000
Diosmetin-7-O-rutin	609	[M+H]+	$C_{28}H_{32}O_{15}$	Flavonoid	16433333	15500000	15033333	22133333	22633333	26100000
Luteolin-7,3'-Di-O-β-D-Glucoside	611	[M+H]+	$C_{27}H_{30}O_{16}$	Flavonoid	14050	45000	68800	26600	24667	63000
Quercetin 3,7-bis-O-β-D-glucoside	627	[M+H]+	$C_{27}H_{30}O_{17}$	Flavonols	49633	138033	120800	140333	68100	216300
Isoorientin	449	[M+H]+	$C_{21}H_{20}O_{11}$	Flavonoid carbonoside	1956667	4390000	5740000	2447000	1030333	3970000
Casticin	375	[M+H]+	$C_{19}H_{18}O_{8}$	Dihydroflavone	20233	17233	17433	16767	12567	9340
Aureusidin	287	[M+H]+	$C_{15}H_{10}O_{6}$	Sinensetin	461333	433333	431333	820000	483000	692000
Wogonin	285	[M+H]+	$C_{16}H_{12}O_{5}$	Flavonoid	4340	4103	4567	10553	14733	14890
Isosakuranetin	287	[M+H]+	$C_{16}H_{14}O_{5}$	Dihydroflavone	92333	53067	63200	179267	109167	96600
Hesperitin	303	[M+H]+	$C_{16}H_{14}O_{6}$	Dihydroflavone	1390000	887333	1722333	3593333	7140000	7060000
Isosinensetin	373	[M+H]+	$C_{20}H_{20}O_{7}$	Flavonoid	2063333	2350000	2426667	2230000	1836667	1440000
Narirutin	581	[M+H]+	$C_{27}H_{32}O_{14}$	Dihydroflavone	17100000	13400000	15933333	23333333	15800000	13835000
Lonicerin	595	[M+H]+	$C_{27}H_{30}O_{15}$	Flavonoid	8200000	11400000	10346667	12820000	10233333	9975000
Neodiosmin	609	[M+H]+	$C_{28}H_{32}O_{15}$	Flavonoid	15200000	16866667	15066667	21100000	21733333	26900000
Sudachiin B	667	[M+H]+	$C_{30}H_{34}O_{17}$	Flavonoid	5716667	2506667	2216667	1200000	923333	1168000
Sudachiin C	667	[M+H]+	$C_{30}H_{34}O_{17}$	Flavonoid	5770000	2530000	2526667	1249000	984667	1192500
Kaempferol-3-neohesperidoside-7-glucoside	757	[M+H]+	$C_{33}H_{40}O_{20}$	Flavonols	75633	94933	82733	78933	71400	83950
Vitexin-2-O-D-glucopyranoside	595	[M+H]+	$C_{27}H_{30}O_{15}$	Flavonoid carbonoside	7730000	7763333	7966667	7190000	6533333	6365000
Kaempferol-3-rutinoside-7-glucoside	757	[M+H]+	$C_{33}H_{40}O_{20}$	Flavonols	72933	77100	84600	77100	69467	80350
Apigenin-6-C-β-D-xyloside-8-C-β-Darabinoside	535	[M+H]+	$C_{25}H_{26}O_{13}$	Flavonoid carbonoside	50067	60967	77667	38500	15150	17050
Hesperetin C-malonylhexoside	549	[M+H]+	$C_{26}H_{28}O_{13}$	Flavonoid	698667	1143333	1130000	785000	299667	221500

				carbonoside						
5-Hydroxyauranetin	389	[M+H]+	$C_{20}H_{20}O_8$	Flavonols	39733333	40333333	40566667	36366667	33500000	29700000
Linarin	593	[M+H]+	$C_{28}H_{32}O_{14}$	Flavonoid	286000	123667	132833	348000	382667	485000
Chrysoeriol-7-O-[β-D-glucuronopyranosyl-(1→ 2)-O-β-D-glucuronopyranoside]	653	[M+H]+	$C_{28}H_{28}O_{18}$	Flavonoid	9	9	12836	40400	34867	31300
Apigenin-7-O-[β-D-glucuronopyranosyl(1→ 2)-O-β-D-glucuronopyranoside)	623	[M+H]+	$C_{27}H_{26}O_{17}$	Flavonoid	2535	10287	16567	9690	4260	1081
6-Hydroxykaempferol-7-O-glucoside	465	[M+H]+	$C_{21}H_{20}O_{12}$	Flavonols	371333	741333	515667	309333	178267	266000
6-Hydroxykaempferol-3,6-O-Diglucoside	627	[M+H]+	$C_{27}H_{30}O_{17}$	Flavonols	52700	54700	57733	77967	103967	107000
6-Hydroxykaempferol-7,6-O-Diglucoside	627	[M+H]+	$C_{27}H_{30}O_{17}$	Flavonols	59133	173500	183233	180367	103667	268700
6-Hydroxykaempferol-3-O-rutin-6-O-glucoside	773	[M+H]+	$C_{33}H_{40}O_{21}$	Flavonols	52800	50533	32100	59067	48267	60500
